# Clinical Remarks on Diphtheria and on Lead Colic

**Published:** 1870-11

**Authors:** Thomas F. Rochester


### ART. II.—*Clinical Remarks on Diphtheria and on Lead Colic*, by Prof. THOMAS F. ROCHESTER, M. D.

REPORTED BY F. BRADNACK, MEMBER OF THE CLASS.

Diphtheria is essentially a blood disease, a general disorder, with local manifestations in the throat, around the tonsils, fauces, and hard palate. It, in some cases, though rarely, travels through the eustachian tube to the ear; in the female it may appear in the vagina, or upon the vulva, but as a general rule the local manifestation is confined to the fauces. Occasionally a cut on the hand or some other portion of the body, will take on a diphtheritic appearance; this fact would indicate that the malady is a blood disorder. The danger in diphtheria is three-fold; (1) the danger of suffocation, should the exudation extend to the trachea; (2) the danger of pyemia, from absorption of the poison; (3) the greatest danger is from prostration by exhaustion. Diphtheria may continue a longer or shorter period; it may, though it seldom does, last a year. A first attack predisposes to a second. In the case of a person greatly prostrated by an eruptive disease, diphtheria is very apt to supervene. Once in a great while a rash accompanies this disease. It is a malady quite distinct from scarlet fever, although attempts have been made to show the contrary. Diphtheria is communicable. It is conveyed by contact, or breathing, or by inhaling the exudations. It is sometimes accompanied by an offensive odor, but not necessarily so. The exudation forms in layers, until it becomes a thick membrane. The sequelæ are sometimes fatal, and are often as much to be feared as the disease. One of these sequelæ is albuminuria; another is paralysis. The latter may be local or general, with impossibility of deglutition. When there is difficult deglutition, it is often a precursor of paralysis. Contrary to popular notion, the exudation and danger are not proportionate; in fact, patients with the largest amount of exudation generally get well. Diphtheria is a treacherous disease. Patients sometimes die from hemorrhage from the bowels. The disease requires very close attention. As regards its treatment, the medication should be general, rather than local. It should also be sustaining. The diet should be nutritious; stimulants are often indicated. Iron is almost always needed. It prevents hemorrhage, and thus closes one avenue of danger. The tincture of the chloride is the most eligible preparation. Lime water is also very efficacious. It dissolves the exudated membrane more quickly than anything else. The membrane should never be torn off, nor should any effort be made to disengage it, unless it comes away spontaneously. An excellent method of local treatment is by vaporization, using lime water. This often proves a valuable plan of treatment, the lime water being resolvent, antiseptic and detergent.

---

*Lead Colic* is induced by exposure to the influence of lead, in some form or other. Some persons are more susceptible to its influence than others. In some individuals the disease is produced after being subjected for a very short period of time to the fumes of lead. Painters, and those engaged indoors in compounding or using lead, are more apt to take the disease than those who use paint out of doors. When paint is mixed with turpentine it is more apt to affect the organism injuriously than when mixed with oil.

As illustrative of the extreme susceptibility of some persons to poisoning by lead, an instance is cited in "Watson's Practice of Medicine," of a lady who suffered an attack of lead colic from sleeping in a room wherein hung a newly-painted birdcage! The poison of lead is often communicated through the medium of water, lead being most frequently found in rain water, this latter being often conveyed through leaden pipes into dwellings and other buildings. In the case of an artisan working with lead in a close room, the poison would be taken both by inhalation, and by absorption through the skin. In this disease, the pain is of an intermittent character, and is greatest at the umbilical region. The pains are those ordinarily defined as colicky. The gums should always be examined for what is known as the "lead line." This line (which is a blue mark around the edge of the gums) is more apt to be seen in persons who are habitually uncleanly, and to whom the tooth brush is an unknown implement. The explanation of this linear discoloration is found in the fact that the sulphuretted hydrogen (generated of uncleanliness) combine chemically with the lead introduced into the system. In persons of scrupulous cleanliness the lead line is not discoverable. In the treatment of this disease, the first indication is to allay the pain by the administration of an anodyne. It is generally bad practice, and also prejudicial to the patient, to give cathartics. There are advantages in giving the anodyne subcutaneously, one of which is the prevention of vomiting. Washing the body will often remove much of the lead, and should never be omitted. After the pain has been quieted by the anodyne, free evacuations will often take place from the bowels, without the use of cathartics, by reason of the general relaxation of the system which has taken place. Should no catharsis occur, castor oil may be administered, or sulphate of magnesia. It is imagined by some that there is a great point gained by giving this latter remedy, it being asserted that the sulphate of magnesia combines chemically with the lead, thus forming an inert salt, but the notion is far-fetched and unsubstantiated; and the castor oil is therefore preferable. In poisoning by lead, when a large quantity has been accidentally swallowed, the sulphate of zinc, combined with ipecachuanha, and used as an emetic, is very advantageous. In this case the sulphate of lead is formed in the stomach, which is not so dangerous as that which it replaced. Afterwards, give sulphate of magnesia. In local poisoning, small doses of iodide of potassium given three times a day act well, the lead passing from the system as iodide of lead. In answer to the question, how can this disease be prevented—what is the best prophylactic treatment—it may be summed up in the word "cleanliness." Let baths be freely used, and garments constantly changed, for the disease is produced far more by absorption than by inhalation of the metal. In some white lead manufactories "sulphuric acid lemonade" is given to the workmen, and, as a prophylactic, often works well.

